# Rationale and Design of the SMILE Registry: A Comprehensive Approach to Predicting Treatment Outcomes in Mitral Regurgitation

**DOI:** 10.3390/jcm15041495

**Published:** 2026-02-14

**Authors:** Myrthe J. M. Welman, Ralph A. L. J. Theunissen, Noa S. A. Wolfs, Sebastian A. F. Streukens, Caroline Jaarsma, Geert Tjeerdsma, Loes P. Hoebers, Peter Luyten, Jindrich Vainer, Patrique Segers, Peyman Sardari Nia, Samuel Heuts, Arnoud W. J. van ‘t Hof, Pieter A. Vriesendorp

**Affiliations:** 1 Department of Cardiology, Maastricht University Medical Centre, 6229 HX Maastricht, The Netherlands; myrthe.welman@mumc.nl (M.J.M.W.);; 2 Cardiovascular Research Institute Maastricht (CARIM), Maastricht University, 6229 ER Maastricht, The Netherlands; 3 Department of Cardiology, Zuyderland Medical Centre, 6419 PC Heerlen, The Netherlands; 4 Department of Cardiology, VieCuri Medical Centre, 5912 BL Venlo, The Netherlands; 5 Department of Cardiology, Laurentius Hospital, 6043 CV Roermond, The Netherlands; 6 Department of Cardiothoracic Surgery, Maastricht University Medical Centre, 6229 HX Maastricht, The Netherlands

**Keywords:** mitral valve regurgitation, multidisciplinary management, treatment outcomes, quality of life, long-term survival, follow-up, registry

## Abstract

**Background**: Mitral valve regurgitation (MR) is associated with an impaired prognosis. Due to its frequently asymptomatic presentation, MR often leads to undertreatment, which can delay diagnosis. In addition, the optimal timing and choice of intervention remain unclear. The Significant Mitral Insufficiency Limburg Evaluation (SMILE) registry aims to provide a comprehensive real-world characterisation of patients with moderate-to-severe mitral regurgitation. **Methods and Results**: The SMILE registry is a multicentre prospective registry initiated in 2020, enrolling all consecutive patients with moderate-to-severe MR from all hospitals in Limburg, the Netherlands. Treatment approaches include surgical, catheter-based, and conservative (medical) options, with decision-making from an expert multidisciplinary team. Data are collected via Castor EDC, ensuring compliance with the General Data Protection Regulation using pseudonymisation. The co-primary endpoints are all-cause mortality and heart failure hospitalisations during the five-year follow-up. Secondary objectives include describing management strategies, characterising disease progression and cardiac remodelling, evaluating associations between baseline clinical and echocardiographic characteristics and long-term outcomes, identifying predictors of treatment success, and assessing longitudinal changes in health-related quality of life. **Conclusions**: The SMILE registry represents an important step towards improving MR management. Its broad data collection, including conservative care and patient-reported outcomes, provides valuable real-world insights beyond procedure-focused studies. The registry may refine intervention timing and personalise treatment strategies to enhance patient outcomes and improve their quality of life.

## 1. Introduction

Mitral valve regurgitation (MR) affects approximately 24 million individuals worldwide and is associated with heart failure, frequent hospitalisations, and increased mortality and morbidity [1,2,3]. The prevalence of moderate-to-severe MR rises with age, affecting 10% of individuals over the age of 75 years [4,5]. Despite its impact, many individuals remain untreated, leading to adverse remodelling and progression to severe disease [3,6,7]. Compensatory cardiac mechanisms may prolong asymptomatic periods, delaying diagnosis and intervention [3,7,8]. Despite MR’s prevalence and potential for favourable recovery, data on its undertreatment are limited [4,9].

In addition, recent advancements in diagnostic and treatment technologies have enhanced clinical outcomes, allowing even complex cases to be managed successfully across varying levels of invasiveness [10]. A multidisciplinary mitral valve team (MDT) is recommended to ensure the optimal management of MR and improve long-term survival outcomes [11,12]. Nevertheless, discrepancies among current guidelines complicate decision-making, leaving key questions unclear, such as the optimal timing and choice for intervention [9,13]. In asymptomatic patients with primary MR (PMR), a ‘watchful waiting’ approach may be appropriate. Conversely, secondary MR (SMR) often requires a more intensive strategy, including regular follow-ups and potentially surgical intervention to enhance long-term survival outcomes [11]. Enhancing characterisation through early recognition, accurate etiological classification, and timely intervention could optimise treatment outcomes and improve the health-related quality of life (HRQoL) for individual patients with severe MR [9].

In the context of asymptomatic severe MR, optimal management remains a topic of debate, largely due to the uncertain long-term outcomes associated with current treatment approaches [3,5]. Without robust clinical trial data, evidence from large multicentre registries is essential. Therefore, the multicentre prospective Significant Mitral Insufficiency Limburg Evaluation (SMILE) aims to evaluate long-term clinical outcomes in patients with moderate-to-severe MR in a real-world setting and to identify clinical and echocardiographic factors associated with disease progression and treatment success. By systematically collecting comprehensive data within an MDT, the registry facilitates a tailored decision-making approach in MR management, aiming to optimise care pathways and assess its impact on HRQoL. Herein, we outline the design of the SMILE registry and describe how all registry procedures are implemented into clinical care.

## 2. Materials and Methods

### 2.1. Registry Population

The SMILE registry is a multicentre prospective registry initiated in 2020. It includes patients with moderate-to-severe MR aged 18 years or older from four hospitals in Limburg, the Netherlands: Maastricht University Medical Centre (MUMC+, Maastricht), Zuyderland Medical Centre (Heerlen/Sittard), Laurentius Hospital (Roermond), and VieCuri Medical Centre (Venlo). The registry protocol was reviewed and approved by the MUMC+ ethics committee ([revision 11115, 2 December 2020]) and adheres to the ethical guidelines outlined in the 1975 Declaration of Helsinki.

### 2.2. Registry Endpoints

The SMILE registry aims to provide a comprehensive, real-world characterisation of patients with moderate-to-severe MR, with co-primary endpoints of all-cause mortality and heart failure hospitalisations during five-year follow-up. Secondary aims are to describe management strategies, evaluate associations between patient characteristics and long-term clinical outcomes, evaluate disease progression, identify factors associated with treatment success, and assess longitudinal changes in HRQoL.

#### 2.2.1. Mortality and Heart Failure Hospitalisations

All-cause mortality, along with heart failure hospitalisations, is monitored during annual follow-up to comprehensively assess survival outcomes, track disease progression, and elucidate the impact of MR on cardiac function and patient prognosis. Along with the HRQoL questionnaire, patients are asked whether they have been admitted for heart failure or undergone mitral valve surgery in the past year. The research team always verifies this information through the patient’s medical records, and dates are recorded.

#### 2.2.2. Intervention Data

The registry captures the mitral valve interventions patients receive, as determined by the expert MDT at MUMC+, which is integrated into standard care and available to all participating centres to ensure the most appropriate approach for each patient. Referral to the MDT occurs only when the patient’s cardiologist considers the MR sufficiently severe. The MDT comprises at least one cardiac surgeon with expertise in mitral valves, an interventional cardiologist experienced in catheter-based mitral valve interventions, and a European Association of Cardiovascular Imaging (EACVI)-certified imaging cardiologist. The team evaluates each individual patient and develops a personalised treatment strategy tailored to their specific mitral valve pathology, anatomical eligibility, comorbidities, overall health background, and personal preferences. Given the multicentre nature of the registry, a range of treatment options is available, including (minimally invasive) mitral valve repair and replacement, transcatheter edge-to-edge repair, percutaneous indirect annuloplasty, and transapical chordal repair or transcatheter mitral valve replacement. These procedures are classified into three categories: surgical interventions, catheter-based procedures, and conservative management. All interventions that do not directly target the mitral valve but may still have an impact are also recorded in the registry. These include device-based approaches, revascularisation procedures, pulmonary vein isolation, or other electrophysiology interventions.

#### 2.2.3. Echocardiographic Assessments

Monitoring longitudinal echocardiographic changes will provide crucial insights into the impact of MR on cardiac mechanics and the heart’s adaptation to treatment. Echocardiographic measurements and MR severity are assessed in accordance with guideline-recommended criteria at the time of analysis [14,15]. Echocardiograms are performed at each participating centre by certified cardiac sonographers and subsequently reviewed by a cardiologist.

Echocardiographic assessment is performed preferentially using transthoracic echocardiography, with transoesophageal echocardiography used if transthoracic echocardiography is unavailable. Baseline patient characteristics and echocardiographic parameters from the most recent echocardiogram are recorded, with follow-up echocardiograms obtained annually if undertaken. In cases where the aetiology of mitral valve disease is unclear, it is recorded as ‘unknown’.

#### 2.2.4. Symptomatic Progression

Symptomatic progression is evaluated using the New York Heart Association (NYHA) functional classification, which provides a standardised measure of the severity of dyspnoea symptoms. At each follow-up visit, patients are assessed by trained clinical staff, and their NYHA class is determined based on patient-reported symptoms and clinical evaluation.

#### 2.2.5. Health-Related Quality of Life

Additionally, HRQoL is assessed at baseline and annually using the 36-Item Short Form Survey (SF-36) by post. This validated questionnaire measures the impact of MR and its treatments on daily activities, emotional well-being, and overall health, providing a view of patient health beyond just the clinical environment. Quality of life changes are stratified based on a minimal clinically important difference of ≥5 points, with changes classified as reduced HRQoL (≥5 points decrease), similar HRQoL (<5 points difference), or increased HRQoL (≥5 points increase), based on a large registry of randomised trials [16].

### 2.3. Registry Enrolment

The registry captures real-world clinical care, with no additional examinations or mandatory follow-ups performed, ensuring that patient management reflects routine practice in the region. Implementing an opt-out system enables patient recruitment without requiring formal written consent, thereby minimising delays and facilitating the inclusion of nearly all eligible participants. Patient inclusion will remain ongoing without a predefined endpoint. All data collected in the SMILE registry are recorded in the electronic Case Report Form [Castor EDC]. The specific data collected in the registry are detailed in the Supplementary Materials. The registry also includes patients who have previously undergone mitral valve interventions. These prior procedures are recorded in the database, and this subgroup may be analysed separately while remaining part of the overall cohort of patients with moderate-to-severe MR.

### 2.4. Data Management and Protection

Patient identifiers are pseudonymised and then entered in coded forms. Tools such as the range and embedded explanatory texts are integrated to guide data entry. Each site is managed by a registry coordinator, who serves as the primary contact for the site and has access to all site-specific data within the EDC. The General Data Protection Regulation is adhered to.

### 2.5. Statistical Considerations and Stratification

Baseline characteristics will be summarised using descriptive statistics. Categorical data will be presented as frequencies and percentages and compared using the Chi-square test. Ordinal variables will be analysed as frequencies and compared using ordinal or non-parametric tests. Continuous data will be reported as means (±standard deviations) or medians (interquartile ranges) based on the distribution, and comparisons will be made using the independent *t*-test for normally distributed data or the Mann-Whitney U test for non-normally distributed data.

#### 2.5.1. Clinical Endpoints

All-cause mortality and time-to-first heart failure hospitalisations will be analysed using Kaplan–Meier estimates and compared using the log-rank test. Associations between baseline characteristics and mortality will be evaluated using multivariable Cox proportional hazards regression models, with results reported as hazard ratios (HRs) with 95% confidence intervals (CIs), using a minimum of 10 events per variable to ensure statistical robustness.

#### 2.5.2. Disease-Specific Endpoints

Longitudinal changes in MR severity will be assessed using mixed-effects models based on annual echocardiographic evaluations. MR severity will be treated as an ordinal outcome, with random intercepts at the patient level to account for within-patient correlation over time. MR progression will be defined as an increase of at least one MR grade from the baseline.

Left ventricular (LV) and left atrial (LA) remodelling will be assessed using linear mixed-effects models applied to continuous echocardiographic parameters, left ventricular end-diastolic diameter (LVEDD) and left ventricular ejection fraction (LVEF), while LA remodelling will be assessed using LA volume index (LAVi). For clinical interpretability, remodelling severity will additionally be categorised according to guideline-recommended cut-off values in secondary analyses. Remodelling severity will be categorised according to established cut-offs as per guideline-defined criteria at the time of assessment [14,15]. For patients already in the most severe category at baseline, progression will be defined as a relative change of ≥10% from the baseline measurement.

#### 2.5.3. Symptom Burden and Health-Related Quality of Life

Symptom burden will be analysed longitudinally using ordinal mixed-effects models. Improvement or worsening of symptoms will additionally be described using dichotomous outcomes, defined as a change of at least one NYHA class relative to the baseline. Health-related quality of life will be analysed using linear mixed-effects models to assess longitudinal changes over follow-up. Additionally, a mediation analysis exploring whether changes in MR severity contribute to improvements in HRQoL will be considered hypothesis-generating.

#### 2.5.4. Treatment Success

Treatment success will be evaluated using responder-based analyses at pre-specified annual landmark time points. For patients undergoing mitral valve intervention, treatment success at each landmark time point will be defined as MR severity ≤2, in combination with an improvement in symptom burden (≥1 NYHA class) and/or HRQoL (SF-36 improvement ≥5 points) compared with the baseline. For patients managed conservatively, treatment success will be defined as clinical stability, characterised by the absence of heart failure hospitalisation or mitral valve intervention during follow-up, together with non-worsening MR severity and stable or improved symptoms or HRQoL.

Associations between baseline characteristics and treatment success will be evaluated using multivariable logistic regression models, with results reported as odds ratios (ORs) and 95% confidence intervals. Left ventricular and LA remodelling parameters will be analysed as parallel longitudinal outcomes and will not be used as sole determinants of treatment success.

#### 2.5.5. Stratification, Exploratory Analyses, and Missing Data

Analyses will be primarily stratified by MR aetiology and, where appropriate, by management strategy (conservative, catheter-based, or surgical interventions). Exploratory analyses will include procedure-specific evaluations and subgroup analyses by MR subtype, which will be classified as PMR or SMR, with further subdivision into ventricular functional MR (VFMR) and atrial functional MR (AFMR) when a sufficient sample size allows. The treatment strategy will be handled as a time-updated exposure, whereby patients contribute person-time to the conservative management group until the occurrence of a mitral valve intervention, after which they are classified in the interventional group. The type of intervention received will be verified for each subgroup, and, once the population size permits, analyses may be conducted for individual intervention types. Patients with a history of prior mitral valve interventions will be clearly identified. Depending on the specific analysis and its relevance, these patients will be included or excluded accordingly. When appropriate, a dedicated subgroup analysis will be performed to specifically evaluate this population.

Missing data will not be imputed to preserve the real-world characteristics of the registry. Primary analyses will be performed using all available data. To evaluate the robustness and consistency of the findings, sensitivity analyses will be conducted in a complete-case cohort to assess potential selection bias.

Covariates will be selected a priori based on clinical relevance, with age and sex included in all models. Univariable analyses will be used to describe crude associations and to inform the selection of additional candidate variables while avoiding purely significance-based screening. Statistical significance will be defined as a two-sided *p*-value < 0.05. Analyses will be performed using RStudio version 2024 or later (RStudio, PBC, Boston, MA, USA).

### 2.6. Interim Analysis of Baseline Characteristics and Early Follow-Up

To date, 476 patients have been enrolled in the SMILE registry, including 238 patients with PMR, 138 with SMR, and 100 with an undetermined aetiology. The latter was attributable to insufficient diagnostic information early in the work-up and to mixed or inconclusive echocardiographic features, resulting in delayed definitive classification. Aetiology classification will be updated during follow-up if additional diagnostic information becomes available. At this interim analysis, the prevalence of atrial fibrillation was significantly higher in patients with SMR compared with those with PMR (PMR: 23.6%; SMR: 39.9%; *p* = 0.003). Patients with PMR exhibited more favourable left ventricular function, reflected by higher LVEF values (PMR: 57.0 [53.9–61.1]%; SMR: 46.0 [35.0–55.0]%; *p* < 0.001) and smaller LVEDD (PMR: 53.3 ± 6.9 mm; SMR: 56.0 ± 8.9 mm; *p* < 0.001) and left ventricular end-systolic diameter (PMR: 36.0 [32.0–40.0] mm; SMR: 44.0 [35.5–51.5] mm; *p* < 0.001). Conversely, a higher proportion of patients in the PMR group presented with MR grade IV (36.6% vs. 19.7%; *p* < 0.001) and had a larger effective regurgitant orifice area (PMR: 0.3 [0.2–0.5] cm^2^; SMR: 0.2 [0.1–0.3] cm^2^; *p* < 0.001). No significant differences were observed in LAVi (PMR: 56.0 [43.5–71.0] mL/m^2^; SMR: 57.4 [46.4–74.0] mL/m^2^; *p* = 0.291).

Baseline HRQoL data were available for 79.0% of patients. Patients with PMR reported significantly higher baseline physical component scores compared with those with SMR (PMR: 70.0 [47.2–88.7]; SMR: 55.6 [40.7–80.6]; *p* = 0.019).

At the time of this interim analysis, 205 patients had reached one-year follow-up, with a questionnaire completion rate of 55.0%; additional responses are pending. One year after enrolment, 44.8% of patients with PMR and 29.1% of patients with SMR were discussed in the multidisciplinary team, of whom 65.4% and 52.2%, respectively, underwent mitral valve intervention. During this early follow-up period, all-cause mortality was 7.8%, and heart failure-related hospitalisation occurred in 9.8% of the total study population.

These results represent an interim, global overview of the baseline characteristics and early follow-up outcomes within the SMILE registry. More detailed, procedure-specific, and outcome-focused analyses will be performed as follow-up accumulates and will be reported in future publications.

## 3. Discussion

The mitral valve has gained considerable attention in recent years due to advances in surgical and catheter-based interventions. Despite these advances, many patients with moderate-to-severe MR remain untreated, largely due to uncertainties surrounding the optimal timing for intervention [3,6,7,8]. The multicentre prospective SMILE registry aims to address these critical gaps by tracking all-cause mortality and heart failure hospitalisations, evaluating disease progression, and identifying predictors of successful MR management.

Previous studies have shown that patients with MR are often referred only at an advanced stage, frequently presenting with severe symptoms, atrial fibrillation, LV systolic dysfunction, or elevated right ventricular systolic pressures. Consequently, only a small percentage of patients receive early intervention, even in patients with PMR [15,16]. Despite its limited use in clinical practice, the American College of Cardiology/American Heart Association (ACC/AHA) guideline suggests that early surgery in PMR may improve outcomes in asymptomatic patients with preserved LV function [7]. Moreover, studies have demonstrated that dedicated MDT discussions contribute to improved survival, emphasizing the importance of structured decision-making in MR management [12]. Most existing registries predominantly focus on patients undergoing mitral valve intervention and therefore capture the selected late-stage populations [17,18,19]. A key strength of the comprehensive, disease-based SMILE registry is its inclusion of all patients with moderate-to-severe MR, including those who do not undergo intervention, those who meet the criteria but are not referred, and those who are not discussed in a multidisciplinary team. By capturing the full disease spectrum, SMILE mitigates selection bias inherent to procedure-based registries, enabling a more accurate description of the natural history of MR, unbiased comparisons across treatment strategies, and evaluation of conservative management. The multicentre nature of the registry enables robust assessment of emerging technologies and management approaches across both academic and non-academic hospitals in Limburg, The Netherlands.

### 3.1. Long-Term Prognosis Across MR Aetiologies

Studies have demonstrated that MR is associated with increased mortality, irrespective of the underlying aetiology [20]. This emphasizes the importance of evaluating long-term outcomes, with five-year overall survival serving as a key indicator of both treatment efficacy and overall disease burden. Understanding how different management strategies influence these outcomes is particularly relevant given that PMR and SMR require distinct approaches. Patients with PMR are often suitable for watchful waiting, whereas those with SMR may need more frequent and prolonged follow-up [11]. Tracking heart failure events separately from non-cardiovascular mortality allows the SMILE registry to capture the nuances of disease progression and the impact of treatment timing. Evidence that specific intervention strategies reduce mortality could inform guideline updates toward earlier or more tailored interventions, while minimal survival benefit despite intervention may underscore the need for optimised patient selection. By systematically evaluating long-term outcomes across both aetiologies, the SMILE registry is well-positioned to provide real-world evidence that addresses these clinical questions.

### 3.2. Phenotyping and Risk Stratification in Mitral Regurgitation

Collecting detailed patient characteristics enables the identification of subgroups that may benefit from specific treatments, supporting more personalised decision-making. The MITRACURE registry demonstrated that only 35% of patients undergoing mitral valve surgery were women [19]. In contrast, the SMILE registry includes patients managed conservatively, allowing for investigation of whether gender influences management and the potential for underdiagnosis or undertreatment.

In the interim analysis, atrial fibrillation was more prevalent in SMR than in PMR, representing an additional clinical observation. This finding aligns with the emerging recognition of AFMR as a distinct phenotype characterised by LA remodelling and preserved LV function, for which favourable outcomes following transcatheter edge-to-edge repair have been reported [21]. The SMILE registry provides an opportunity to further characterise this phenotype within routine clinical practice.

Beyond clinical phenotyping, echocardiographic assessment remains fundamental to evaluating disease progression and treatment response. Identifying parameters consistently associated with favourable outcomes may support their integration into routine care and guide clinical decision-making. Prior research has demonstrated that certain echocardiographic metrics are linked to differences in mortality [20]. By capturing these data longitudinally, SMILE can identify the parameters most informative for MDT discussions, thereby supporting patient-specific management decisions aimed at optimising survival.

### 3.3. Functional Status and Quality of Life in Routine Practice

The NYHA class is widely used to guide the timing of mitral valve interventions. Current ACC/AHA guidelines recommend surgery or transcatheter intervention for symptomatic patients with secondary severe MR (NYHA class III or IV), despite optimal GDMT [7]. NYHA class is also often a key criterion in clinical trials and independently predicts mortality and adverse outcomes. However, it has limitations, including interobserver variability and reduced sensitivity, meaning that patients classified as NYHA I or II may still experience significant reductions in HRQoL [22]. The SF-36 is an established tool for assessing HRQoL across eight health domains, including physical functioning, pain, general health, vitality, social functioning, emotional functioning, and mental health. Due to the use of the SF-36 questionnaire, the SMILE registry provides a more comprehensive evaluation, enabling the identification of patients whose health status may be worse than indicated by their NYHA classification.

Surgical mitral valve repair or replacement has been shown to improve disease-specific HRQoL, particularly in patients with poor baseline function [23,24,25]. Less invasive approaches, including minimally invasive surgery and transcatheter mitral valve repair, also offer an improvement in HRQoL [23,26]. Importantly, these findings are mostly derived from patients who underwent intervention, while data on conservatively managed or late-referred patients are limited. The SMILE registry addresses this gap by systematically collecting HRQoL data in all patients with moderate-to-severe MR, enabling comparisons between treatment modalities, evaluation of long-term well-being, and support for clinical decision-making. By integrating patient-reported outcomes with clinical parameters, SMILE can identify patients who may benefit from earlier intervention and inform personalised treatment strategies. However, while SF-36 is a robust tool, its subjective nature and potential demographic biases should be interpreted alongside clinical data to fully assess treatment effectiveness.

### 3.4. Limitations

The SMILE registry aims to reflect current clinical practices, but due to its multicentre design, not all participating centres acquire the same echocardiographic parameters. The availability of specific measurements may vary according to local expertise, workflow, and clinical priorities. In addition, MR represents a complex and heterogeneous disease, and advanced quantitative parameters such as effective regurgitant orifice area and proximal isovelocity surface area cannot always be reliably assessed in every patient, resulting in missing data. Although guideline-recommended criteria are applied, some degree of interobserver variability is inevitable. Nevertheless, the longitudinal assessment of multiple echocardiographic parameters over a five-year follow-up period allows for the evaluation of within-patient trends rather than a reliance on single measurements, thereby facilitating the identification of inconsistent findings and clinically implausible changes over time. While no formal inter- or intraobserver variability analyses were performed, a cardiologist’s review of echocardiograms as part of routine clinical care may provide quality oversight consistent with real-world practice.

Another limitation of the SMILE registry is that the registry follows patients for five years, a period chosen to balance clinical relevance and feasibility. While this may be insufficient to capture the full natural history in asymptomatic patients or those with PMR, it provides valuable insights into disease progression and treatment outcomes and provides the groundwork for future extended follow-up studies. Finally, the SMILE registry does not systematically record the use of guideline-directed medical therapy (GDMT) or cardiac resynchronisation therapy (CRT), nor does it include routine right heart catheterisation. Both GDMT and CRT are essential in SMR management, improving survival, reducing heart failure events, and promoting reverse remodelling [7]. Right heart catheterisation remains the gold standard for diagnosing pulmonary hypertension, a key prognostic marker, and echocardiographic estimates may underestimate its prevalence and impact [7,27]. Despite these limitations, the SMILE registry provides valuable, long-term follow-up and extensive data on HRQoL in MR patients, which, to our knowledge, is scarce in comparable studies. This unique focus contributes to a deeper understanding of treatment effects on both clinical outcomes and patients’ daily lives, ultimately enhancing MR management quality.

### 3.5. Future Perspectives

Initially, patient consent was required due to the SF-36 questionnaire. As this is a standard, non-invasive measure and no additional procedures will be performed, the registry has operated under an opt-out framework since 2023, approved by the local Medical Ethics Committee. The early logistical challenges delayed full efficiency, but by following local clinical care pathways, inclusion has proceeded smoothly, minimising disruption and ensuring optimal performance. As a comprehensive real-world dataset, the registry is well-positioned to support future studies and randomised clinical trials. Its detailed, longitudinal data on a diverse MR patient population facilitates the investigation of treatment variations and advanced methodologies. In the interim analysis of the SMILE registry, substantial heterogeneity in patient characteristics, disease severity, and management strategies was observed, underscoring the complexity of treatment for moderate-to-severe MR. Therefore, in the future, the robust SMILE data may enable the identification of key predictors of treatment success and survival, refining patient selection criteria for future trials, and guiding the design of more targeted randomised clinical trials. Prospectively, the SMILE registry has the potential for worldwide collaborations with diverse specialities, including cardiac imaging and electrophysiology. These cross-disciplinary collaborations could contribute to the development of new biomarkers and imaging parameters, offering improved predictive accuracy for MR progression. Furthermore, data sharing with international registries and research networks can support worldwide initiatives aimed at improving MR patient care. As the registry continues to evolve, it can contribute to developing evidence-based clinical guidelines, supporting standardised, data-driven MR management. By connecting real-world data with clinical practice within a rigorous, evidence-based framework, the registry offers a valuable opportunity to shape the future of personalised patient care. Ultimately, the insights from the SMILE registry may help inform individual clinical decision-making, optimise patient outcomes, and inform the development of next-generation therapies for MR.

## 4. Conclusions

The SMILE registry has the potential to advance MR management by identifying key predictors of successful treatment and improving our understanding of disease progression. By evaluating clinical parameters, including all-cause mortality and heart failure hospitalisations, as well as health-related quality of life in both treated and untreated patients, SMILE will provide a comprehensive real-world view of MR. Importantly, the inclusion of conservatively managed patients will generate insights beyond procedure-focused studies, supporting the refinement of intervention timing and the development of more personalised treatment strategies aimed at improving patient outcomes and quality of life.

## Data Availability

The data presented in this study are available on request from the corresponding author.

## Supplementary Materials

The following supporting information can be downloaded at: https://www.mdpi.com/article/10.3390/jcm15041495/s1, The supplementary data contain the full registry protocol of the SMILE registry, along with a comprehensive overview of the collected clinical and echocardiographic data.

## Abbreviations

The following abbreviations are used in this manuscript:

AFMR	Atrial functional mitral regurgitation
CRT	Cardiac resynchronization therapy
GDMT	Guideline-directed medical therapy
HRQoL	Health-related quality of life
LA	Left atrium
LAVi	Left atrial volume index
LV	Left ventricle
LVEDD	Left ventricular end-diastolic diameter
LVEF	Left ventricular ejection fraction
MDT	Multidisciplinary mitral valve team
MR	Mitral regurgitation
NYHA	New York Heart Association
PMR	Primary mitral regurgitation
SF-36	36-Item Short Form Survey
SMILE	Significant Mitral Insufficiency Limburg Evaluation
SMR	Secondary mitral regurgitation
VFMR	Ventricular functional mitral regurgitation
