# Supplementary material for: Rationale and Design of the SMILE Registry: A Comprehensive Approach to Predicting Treatment Outcomes in Mitral Regurgitation
Source: J Clin Med. 2026 Feb 14;15(4):1495. doi: 10.3390/jcm15041495 (PMC12941851; doi:10.3390/jcm15041495)
Supplement: Supplementary file 1 [file jcm-15-01495-s001.zip › jcm-4046106-supplementary.pdf]

Study protocol

The Significant Mitral Insufficiency Limburg Evaluation: a disease-based registry of patients with moderate -severe mitral valve regurgitation (SMILE)

Maastricht, 14 February 2026

**Title**	The Significant Mitral Insufficiency Limburg Evaluation: a disease-based registry of patients with moderate-severe mitral valve regurgitation
**Acronym**	SMILE
**Classification**	Academic, Investigator-initiated registry
**Version**	1.2
**Protocol date**	04-02-2025
**Study initiation**	01-12-2020
**Study duration**	Minimum of 10 years
**Sample size**	All patients diagnosed with moderate-severe mitral regurgitation by echocardiography at participating centres will be included in the study. There will be no predefined stopping point for patient inclusion. These patients will be registered at four participating hospitals and followed for a period of five years.
**Principal Investigators**	PAV/AH
**Coordinating Investigator**	MW
**Steering committee**	PAV/AH/RT/BS/MW
**Study Centres**	Maastricht UMC+, Maastricht, The Netherlands<br>Zuyderland MC, Sittard and Heerlen, The Netherlands<br>VieCuri MC, Venlo, The Netherlands<br>Laurentius Hospital, Roermond, The Netherlands
**Study Hypothesis**	Through the repeated quantitative evaluation of patients with moderate-severe mitral valve regurgitation, we can achieve a more comprehensive

	characterization of disease progression, thereby enabling the individualized adjustment of personalised treatment strategies.
**Study Design**	An observational prospective database will be used. No randomization will take place in this study.
**Study population**	All patients with moderate-severe mitral valve regurgitation, as determined by echocardiography, from the designated participating centres.
**Objectives**	The co-primary endpoints are all-cause mortality and heart failure hospitalisations over a five-year follow-up period. Secondary objectives are to describe management strategies, evaluate mitral regurgitation progression and cardiac remodelling, investigate associations between baseline clinical and echocardiographic characteristics and long-term outcomes, identify predictors of treatment success, and assess longitudinal changes in health-related quality of life.
**Inclusion criteria**	All patients aged 18 years or older with moderate-severe MR as determined by echocardiography.
**Exclusion criteria**	Patients who object to participation in the registry.
**Follow-up**	Five years of follow-up, with an annual collection of patient characteristics, echocardiographic parameters, and the Short Form 36 Health Survey Questionnaire.
**GCP and data protection**	These studies will be performed in compliance with good clinical practices guidelines, and data will be handled in accordance with the European General Data Protection Regulation.
**Confidentiality status**	The information contained herein is confidential and the proprietary property of the principal investigators.

Table of contents

1 Introduction and rationale 8

2 Objectives 9

2.1 Primary objective 9

2.2 Secondary objectives 9

3 Study design 9

3.1 Proposed timeline of trial 9

4.1 Population 10

4.2 Inclusion Criteria 10

4.3 Exclusion Criteria 10

4.4 Sample size 10

4.5 Study flow diagram 11

5 Methods 12

5.1 Study parameters/endpoints 12

5.2 Randomisation, blinding and treatment allocation 12

5.3 Study procedures 12

5.4 Schedule of assessment 12

5.5 Withdrawal of individual subjects 13

5.6 Premature termination of the study 13

5.7 Feasibility of the recruitment schedule 13

6 Safety 14

6.1 Temporal halt for reasons of subject safety 14
6.2 Safety monitoring..... 14
6.3 (Serious) Adverse events and suspected, unexpected severe adverse reactions 14
6.4 Annual safety report..... 14
6.5 Data monitoring safety board..... 15
7 Statistical analyses 16
7.1 Baseline, primary, secondary outcomes..... 16
8 Ethical considerations 17
8.1 Regulation statement..... 17
8.2 Recruitment and consent..... 17
9 Administrative aspects, monitoring and publication..... 18
9.1 Handling of storage and data documents 18
9.2 Amendments 18
10 Risk analysis 19
11 Endpoint definitions..... 20
11.1 Clinical endpoints (Primary) 20
11.2 Disease-specific endpoints (Secondary) 20
11.3 Symptom burden and health-related quality of life (Secondary) 20
11.4 Treatment success (Secondary)..... 21
11.5 Safety outcomes 21
12 References..... 22

13 Appendix 23

List of abbreviations and relevant definitions

**CTCM**	Clinical Trial Center Maastricht
**DSMB**	Data Safety Monitoring Board
**EROA**	Effective regurgitant orifice area
**GDPR**	General Data Protection Regulation
**HR**	Hazard ratio
**HRQoL**	Health-related quality of life
**LA**	Left atrium
**LAVi**	Left atrial volume index
**LV**	Left ventricle
**LVEDD**	Left ventricular end-diastolic diameter
**LVEF**	Left ventricular ejection fraction
**LVESD**	Left ventricular end-systolic diameter
**MR**	Mitral regurgitation
**MUMC+**	Maastricht University Medical Centre+
**NYHA**	New York Heart Association
**PISA**	Proximal isovelocity surface area
**SF-36**	36-Item Short Form Survey
**sPAP**	Systolic pulmonary artery pressure
**SMILE**	Significant Mitral Insufficiency Limburg Evaluation
**WGBO**	Dutch Civil Code: Medical Treatment Contracts Act
**WMO**	Medical Research Involving Human Subjects Act

1 Introduction and rationale

Mitral regurgitation (MR) affects more than 10% of individuals aged 75 years and older and is associated with adverse cardiovascular outcomes, including heart failure, increased hospitalizations, and mortality (1-3). Despite its prevalence, MR is often undertreated, leading to progressive disease, adverse cardiac remodelling, and diminished quality of life (HRQoL) (1, 4, 5). While advancements in diagnostic and therapeutic technologies have improved outcomes, optimal management strategies, particularly in asymptomatic patients, remain unclear due to discrepancies in guidelines and a lack of robust data (6-8). However, timely intervention has been shown to improve outcomes significantly (6).

The multicentre prospective Significant Mitral Insufficiency Limburg Evaluation (SMILE) registry aims to evaluate long-term outcomes in patients with moderate-severe MR in routine clinical practice, and to determine clinical and echocardiographic predictors of MR progression and treatment success. Through a multidisciplinary approach, the registry aims to align the progression of mitral valve disease and care pathways with individual patient needs, optimize treatment strategies, and enhance long-term outcomes, including survival and HRQoL.

2 Objectives

2.1 Primary objective

To evaluate the co-primary clinical endpoints during a five-year follow-up:

- all-cause mortality
- heart failure hospitalisations

2.2 Secondary objectives

- To describe contemporary management strategies in patients with moderate-severe MR.
- To evaluate associations between baseline patient characteristics and long-term clinical outcomes.
- To evaluate MR disease progression over time.
- To identify key predictors associated with treatment success.
- To assess longitudinal changes in HRQoL.

3 Study design

3.1 Proposed timeline of trial

This study aims to create an ongoing, continuous database that will capture all patients with moderate-severe MR in Limburg, allowing for comprehensive long-term data collection and monitoring of this population.

4 Study population

4.1 Population

All patients with moderate-severe MR, as determined by echocardiography, from the designated participating centres (Maastricht University Medical Centre+ (MUMC+), Maastricht; VieCuri Medical Centre, Venlo; Laurentius Hospital, Roermond; Zuyderland Medical Centre, Heerlen, and Sittard/Geleen).

4.2 Inclusion Criteria

All patients aged 18 years and older with moderate-severe MR as determined by echocardiography.

4.3 Exclusion Criteria

Patients who object to participation in the registry.

4.4 Sample size

There will be no predefined endpoint for patient inclusion. The register strives to enrol all patients who meet the criteria for moderate-severe MR, as determined by echocardiography, from the designated participating centres. These patients will be registered at the four participating hospitals and followed up for a period of five years.

4.5 Study flow diagram

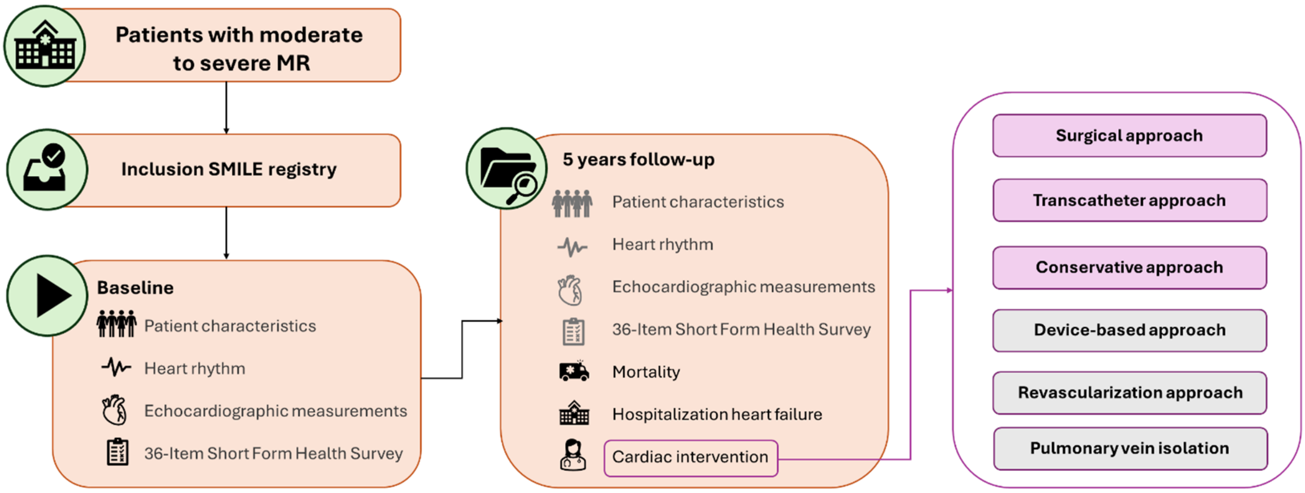

5 Methods

5.1 Study parameters/endpoints

The study included key patient characteristics, such as age, gender, height, weight, and New York Heart Association (NYHA) class. Echocardiographic parameters included left ventricular ejection fraction (LVEF), mitral valve aetiology, MR severity, left atrial volume index (LAVi), effective regurgitant orifice area (EROA), regurgitant volume, and systolic pulmonary artery pressure (sPAP). Follow-up data, including all-cause mortality and heart failure hospitalisations, are collected during annual follow-up and verified through medical records, with dates recorded. The registry additionally captures mitral valve interventions and other cardiac procedures that may influence outcomes. HRQoL is assessed at baseline and annually using the 36-Item Short Form Survey (SF-36). Additional variables, including detailed echocardiographic measurements (e.g., vena contracta, PISA radius, LVEDD, LVESD) and comprehensive follow-up variables (e.g., cardiac interventions), are presented in the appendix.

5.2 Randomisation, blinding and treatment allocation

There is no randomization involved. Participants will continue to receive standard care, and no additional interventions or investigations will be applied.

5.3 Study procedures

The database does not require any changes to the participants' usual care, and they will not undergo any procedures other than echocardiographic evaluation and follow-up. Follow-up will take place through regular outpatient visits at the referring hospital. Participating patients will be asked to complete an annual SF-36 questionnaire, with follow-up occurring once yearly for five years. Completing the SF-36 questionnaire will take approximately 15 minutes. Additionally, data from patient records will be collected for five years.

5.4 Schedule of assessment

No additional procedures are planned. All assessments will follow the standard care protocol.

5.5 Withdrawal of individual subjects

The participants can withdraw from the SMILE registry at any time and are not required to provide a reason for discontinuing their participation. The data collected up to that point will be used for the research.

5.6 Premature termination of the study

Participants may withdraw from the study at any time, either at their request or if the MUMC, the governing authorities, or the ethics review committee decides to terminate the study.

5.7 Feasibility of the recruitment schedule

The recruitment for the SMILE registry is designed to be feasible and efficient within the clinical workflow. Patients with moderate-severe MR are identified during routine echocardiographic examinations. These patients are subsequently contacted via post or telephone to inform them about the registry and their eligibility to participate. The use of an opt-out/no-objection system ensures that recruitment can proceed without the need for formal written consent, reducing delays and enabling the inclusion of nearly all eligible patients. This approach allows for a comprehensive and representative dataset while respecting patient autonomy and privacy.

6 Safety

6.1 Temporal halt for reasons of subject safety

As no additional procedures are performed for the study and standard care is followed, there are no risks associated with participation, and therefore, a safety halt is not applicable.

6.2 Safety monitoring

As the study involves only standard care without additional interventions, comprehensive safety monitoring or the establishment of a Data Safety Monitoring Board (DSMB) is not deemed necessary; however, Clinical Trial Center Maastricht (CTCM) manages the Castor database to ensure proper data handling and regulatory compliance.

6.3 (Serious) Adverse events and suspected, unexpected severe adverse reactions

This study follows standard clinical care procedures, and no additional interventions are introduced. Therefore, no specific adverse events or suspected, unexpected severe adverse reactions related to the study are expected. Any adverse event will be considered part of the standard course of care and will be documented in the patient's medical records as per routine clinical practice. These events will not be specifically attributed to the study, and no additional reporting beyond standard care requirements is deemed necessary.

6.4 Annual safety report

Since this study involves only standard care without additional interventions, there are no study-related risks or adverse events to report. Consequently, the preparation of an annual safety report is not applicable.

6.5 Data monitoring safety board

As the study involves only standard care without additional interventions, comprehensive safety monitoring or the establishment of a DSMB is not deemed necessary; however, CTCM manages the Castor database to ensure proper data handling and regulatory compliance.

7 Statistical analyses

7.1 Baseline, primary, secondary outcomes

Analysis will begin with descriptive statistics to summarise baseline characteristics. Categorical variables will be presented as counts and percentages and compared using the Chi-square test. Ordinal variables will be reported as frequencies and compared using ordinal or non-parametric tests. Continuous variables will be presented as means (± standard deviations) or medians (interquartile ranges), depending on the distribution, and compared using the independent t-test for normally distributed data or the Mann–Whitney U test for non-normally distributed data.

Time-to-event outcomes, including all-cause mortality and time-to-first heart failure hospitalisation, will be analysed using Kaplan–Meier estimates and compared using the log-rank test. Associations between baseline characteristics and clinical outcomes will be evaluated using multivariable Cox proportional hazards regression models, with results presented as hazard ratios (HRs) and 95% confidence intervals (CIs). A minimum of 10 events per variable will be considered to ensure statistical robustness.

Longitudinal changes in echocardiographic parameters and HRQoL will be analysed using mixed-effects models to account for repeated measurements within patients. MR severity will be demonstrated as an ordinal outcome, while continuous echocardiographic measures and HRQoL outcomes will be analysed using linear mixed-effects models. Treatment success will be evaluated using responder-based analyses based on annual follow-up assessments, and predictors of treatment success will be analysed using multivariable logistic regression models, with results presented as odds ratios (ORs) and 95% CIs. Analyses will be stratified by MR aetiology and, where appropriate, by management strategy (conservative, catheter-based, or surgical). Missing data will not be imputed; primary analyses will be conducted using all available data, with sensitivity analyses performed in complete-case cohorts. Statistical significance will be set as a two-sided p-value < 0.05.

8 Ethical considerations

8.1 Regulation statement

The SMILE registry is classified as a non-WMO study and complies with the General Data Protection Regulation and the Dutch Civil Code (WGBO). Under Article 458 of the WGBO, patient data for scientific research without explicit consent is permitted when obtaining such consent is impractical or unreasonable, if patient privacy is safeguarded, and the research serves a legitimate public interest. The ethical framework for this registry has been reviewed and approved by the Medical Ethics Review Committee of Maastricht UMC+.

8.2 Recruitment and consent

The SMILE registry operates under a no-objection system. Patients are eligible for inclusion when moderate-severe MR is observed on a cardiac echocardiogram. These eligible patients are informed about the study through communication via post or telephone, and they are provided with detailed information regarding the purpose and scope of the registry. Patients are explicitly allowed to object to the use of their data. If no objection is raised, their data will be included in the registry. This method ensures compliance with ethical principles while facilitating a comprehensive and unbiased collection of data necessary to evaluate and improve the quality of care for MR patients.

9 Administrative aspects, monitoring and publication

9.1 Handling of storage and data documents

The research data collected by the participating centres will be centrally managed by the research team at the MUMC+. The data will be securely saved, and only coded patient information will be used for data analysis. Access to the data will be restricted to the same research team and will be utilized exclusively within the scope of this study. This is a multicentre registry, where each referring centre records the required patient data in an electronic case report form, managed by CTCM. Each participating centre has a designated local principal investigator responsible for overseeing the data collection process.

9.2 Amendments

An amendment to the SMILE registry protocol was submitted on July 5, 2022, to the Medical Ethics Review Committee of Maastricht UMC+. This amendment proposed the implementation of a no-objection system for patient inclusion, replacing the requirement for written informed consent. The change was necessary to ensure the feasibility and comprehensiveness of patient recruitment since the original consent procedure limited the inclusion rate and the registry's value. The amendment was approved after submission, ensuring compliance with ethical and legal guidelines while enabling the collection of essential data to improve care for MR patients.

10 Risk analysis

This observational study follows standard clinical care protocols, and no additional invasive procedures or treatments are required for participants. All data collection is based on routine clinical evaluations, including echocardiography and annual follow-up visits. Participants are asked to complete a standardised SF-36 questionnaire annually, which is non-invasive and estimated to take approximately 15 minutes.

The primary risk associated with the study is the minimal burden of time and effort required to complete the annual questionnaire and attend follow-up visits. The collection of clinical data from medical records does not introduce any physical risk to participants. Given the observational nature of this study, no additional risks are introduced to participants beyond those inherent to standard clinical care.

11 Endpoint definitions

11.1 Clinical endpoints (Primary)

The primary clinical endpoints are all-cause mortality and time-to-first heart failure hospitalisation during long-term follow-up. Mortality and hospitalisation events will be collected during annual follow-up and, when applicable, verified through medical records with corresponding dates recorded.

11.2 Disease-specific endpoints (Secondary)

MR disease progression will be assessed using longitudinal echocardiographic evaluations performed during routine clinical care. MR severity will be assessed according to guideline-recommended criteria at the time of analysis. MR progression will be defined as an increase of at least one MR grade compared with baseline.

In addition, left ventricular (LV) and left atrial (LA) remodelling will be assessed longitudinally using echocardiographic parameters. LV remodelling will be evaluated using LV end-diastolic diameter (LVEDD) and left ventricular ejection fraction (LVEF), while LA remodelling will be evaluated using LA volume index (LAVi). For clinical interpretability, remodelling severity will additionally be categorised based on guideline-recommended cut-off values at the time of assessment. For patients already in the most severe category at baseline, progression will be defined as a relative change of ≥10% from baseline.

11.3 Symptom burden and health-related quality of life (Secondary)

Symptom burden will be evaluated using the NYHA functional classification at baseline and during follow-up. Symptomatic improvement or worsening will be defined as a change of at least one NYHA class compared with baseline.

Health-related quality of life will be assessed at baseline and annually using the SF-36 to evaluate the impact of MR and its treatment on daily activities and overall well-being. Longitudinal changes in HRQoL will be interpreted using a minimal clinically important difference of ≥5 points (improvement or deterioration).

11.4 Treatment success (Secondary)

Treatment success will be evaluated using responder-based analyses derived from annual follow-up assessments. For patients undergoing mitral valve intervention, treatment success will be defined as the first follow-up assessment at which MR severity is ≤2, in combination with improvement in symptom burden (≥1 NYHA class) and/or improvement in HRQoL (SF-36 increase ≥5 points) compared with baseline. For patients managed conservatively, treatment success will be defined as clinical stability, characterised by the absence of heart failure hospitalisation or mitral valve intervention during follow-up, together with non-worsening MR severity and stable or improved symptoms and/or HRQoL. LV and LA remodelling parameters will be analysed as longitudinal outcomes but will not be used as sole determinants of treatment success.

11.5 Safety outcomes

No safety outcomes are defined for this study, as it is observational and involves no additional interventions beyond standard care.

12 References

1. Dziadzko V, Clavel M-A, Dziadzko M, Medina-Inojosa JR, Michelena H, Maalouf J, et al. Outcome and undertreatment of mitral regurgitation: a community cohort study. The Lancet. 2018;391(10124):960-9.

2. Baskett RJF, Exner DV, Hirsch GM, Ghali WA. Mitral insufficiency and morbidity and mortality in left ventricular dysfunction. Canadian Journal of Cardiology. 2007;23(10):797-800.

3. Nkomo VT, Gardin JM, Skelton TN, Gottdiener JS, Scott CG, Enriquez-Sarano M. Burden of valvular heart diseases: a population-based study. The Lancet. 2006;368(9540):1005-11.

4. Sparano DM, Ward RP. Management of asymptomatic, severe mitral regurgitation. Curr Treat Options Cardiovasc Med. 2012;14(6):575-83.

5. Otto CM, Nishimura RA, Bonow RO, Carabello BA, Erwin JP, Gentile F, et al. 2020 ACC/AHA Guideline for the Management of Patients With Valvular Heart Disease: A Report of the American College of Cardiology/American Heart Association Joint Committee on Clinical Practice Guidelines. Circulation. 2021;143(5):e72-e227.

6. Suri RM, Vanoverschelde J-L, Grigioni F, Schaff HV, Tribouilloy C, Avierinos J-F, et al. Association between early surgical intervention vs watchful waiting and outcomes for mitral regurgitation due to flail mitral valve leaflets. Jama. 2013;310(6):609-16.

7. Iung B, Delgado V, Rosenhek R, Price S, Prendergast B, Wendler O, et al. Contemporary Presentation and Management of Valvular Heart Disease: The EURObservational Research Programme Valvular Heart Disease II Survey. Circulation. 2019;140(14):1156-69.

8. Galusko V, Sekar B, Ricci F, Wong K, Bhattacharyya S, Mullen M, et al. Mitral regurgitation management: a systematic review of clinical practice guidelines and recommendations. Eur Heart J Qual Care Clin Outcomes. 2022;8(5):481-95.

9. Contopoulos-Ioannidis DG, Karvouni A, Kouri I, Ioannidis JP. Reporting and interpretation of SF-36 outcomes in randomised trials: systematic review. Bmj. 2009;338:a3006.

13 Appendix

Category	Variable	Type	Details
**Baseline Characteristics**	Date of visit	Date	Date of the initial visit
	Gender	Categorical	Male, Female
	Age at baseline	Numeric	Age in years at the time of the initial visit
	Height	Numeric	Height in centimetres
	Weight	Numeric	Weight in kilograms
	NYHA (Dyspnea class)	Categorical	Dyspnea class (I, II, III, IV)
	Heart rhythm	Categorical	Sinus rhythm, Atrial fibrillation, Atrial flutter, Pacemaker rhythm, Other
	Heart rate	Numeric	Heartbeats per minute
**Baseline and Follow-up Echocardiographic Parameters**	Date of echocardiography	Date	Date of the echocardiography
	LVEF (%)	Numeric	Left ventricular ejection fraction
	Aetiology	Categorical	Primary, Secondary
	Flail leaflet	Categorical	Yes, no
	Involved segment in mitral valve prolapse	Categorical	P1, P2, P3, A1, A2, A3, Posteromedial commissure, Anterolateral commissure

	Severity of MI	Categorical	Mild (Grade I), Mild to moderate (Grade II), Moderate to severe (Grade III), Severe (Grade IV)
	ERO	Numeric	Effective regurgitant orifice
	LAVi	Numeric	Left atrial volume index
	Regurgitant volume	Numeric	Volume of regurgitation
	E/A ratio	Numeric	Ratio of E-wave to A-wave
	Maximum E-wave velocity	Numeric	Maximum velocity of E-wave
	Vena contracta	Numeric	Diameter of vena contracta
	PISA radius	Numeric	Proximal Isovelocity Surface Area
	LVEDD	Numeric	Left ventricular end-diastolic diameter
	LVESD	Numeric	Left ventricular end-systolic diameter
	sPAP	Numeric	Systolic pulmonary artery pressure
	Pulmonary vein backflow	Categorical	Yes, no
	A-wave dominant mitral inflow	Categorical	Yes, no
	Tricuspid regurgitation	Categorical	None, Trace, Mild, Moderate, Severe
	Aortic valve stenosis	Categorical	None, Mild, Moderate, Severe
**Follow-up at 1 Year**	Date of visit	Date	Date of the follow-up visit
	Age at 1-year follow-up	Numeric	Age in years at the time of the 1-year follow-up

	Height	Numeric	Height in centimetres
	Weight	Numeric	Weight in kilograms
	NYHA (Dyspnea class)	Categorical	Heart failure class (I, II, III, IV)
	Heart rhythm	Categorical	Sinus rhythm, Atrial fibrillation, Atrial flutter, Pacemaker rhythm, Other
	Heart rate	Numeric	Heartbeats per minute
	Mortality	Categorical	Yes, no
	Heart failure hospitalization	Categorical	Yes, no
	Cardiac interventions	Categorical	Transcatheter Edge-to-Edge Repair, Mitral Valve Replacement, Mitral Valve Repair, Tendyne Transcatheter Mitral Valve Replacement system, Neochord Transapical Artificial Chordae Repair system, Carillon Mitral Contour System (percutaneous indirect annuloplasty), Percutaneous Coronary Intervention, Coronary Artery Bypass Grafting, Aortic Valve Replacement, Permanent Pacemaker implantation, Implantable Cardioverter-Defibrillator implantation, Pulmonary Vein Isolation, None
